# Do Symbiotic Microbes Drive Chemical Divergence Between Colonies in the Pratt’s Leaf-Nosed Bat, *Hipposideros pratti*?

**DOI:** 10.3390/biology15020114

**Published:** 2026-01-06

**Authors:** Ziqi Zheng, Jeffrey R. Lucas, Chunmian Zhang, Congnan Sun

**Affiliations:** 1 Hebei Key Laboratory of Animal Physiology, Biochemistry and Molecular Biology, College of Life Sciences, Hebei Normal University, Shijiazhuang 050024, China; ziqizheng@stu.hebtu.edu.cn; 2 Hebei Collaborative Innovation Center for Eco-Environment, Hebei Normal University, Shijiazhuang 050024, China; 3 Department of Biological Sciences, Purdue University, West Lafayette, IN 47907, USA; jlucas@purdue.edu

**Keywords:** bats, chemical signals, geographical variation, microbiota

## Abstract

Host odour may be affected by symbiotic microbes that produce metabolites. As a result, chemical signal production may be influenced. Few studies to date have assessed how symbiotic microbes influence variation in geography of animal chemical signals. This is important because chemical signal divergence can affect mate choice, species recognition, and ultimately speciation in a broad range of animals. However, the underlying driving forces of chemical signal divergence are still rather poorly understood. To study chemical signals, bats provide a good model system because they are such social mammals. Because males roost in dark spaces during the daytime, they rely on chemical and acoustic signals. We identified three colonies across a large geographic area and collected male forehead gland secretions from Pratt’s leaf-nosed bats (*Hipposideros pratti*). We examined the role symbiotic microbes played in potential variation in the geography of chemical signals. We observed significant colony-level differences in compound categories and in the amount of specific compounds. We also found significant colony-level differences in forehead gland microbiota. However, there was no significant relationship between bat-gland bacterial community composition and variation in chemical composition across colonies. These results suggest that bacterial communities may fail to shape the chemical signalling profiles of the different colonies in Pratt’s leaf-nosed bats.

## Simple Summary

Animals’ chemical signals often exhibit geographical variation. Previous studies on the potential driving forces influencing chemical signal divergence concentrate on ecological selection and genetic drift of the signals per se; the potential for symbiotic microbes to influence chemical signal divergence has received far less attention. Here, we studied Pratt’s leaf-nosed bat, *Hipposideros pratti*, to examine whether the symbiotic microbes inhabiting their odour glands may explain the observed chemical variation between colonies. Chemical signals from the forehead gland secretions of male *H. pratti* across three colonies demonstrated significant variation in geography. However, chemical signal divergence was not significantly correlated with geographic variation in the forehead gland microbiota. According to these results, symbiotic microbes may not be essential in the divergence of chemical signals among in *H. pratti*. Our work not only expands upon research into the geographical variation in chemical signals in bats but also provides theoretical support for subsequent inquiries into bat speciation processes, ultimately offering a scientific basis for protecting bat diversity.

## 1. Introduction

Chemical signals can represent important phenotypes of animals, playing a crucial role in their survival and reproduction, especially in insects and mammals [1]. Moreover, chemical signals often exhibit geographical variation [2]. Variation in geography among chemical signals may have an impact on the ability of different populations of a species to recognize each other. As a result, reproductive isolation, assortative mating, and speciation may be affected [2]. To better understand the mechanisms of population differentiation and speciation, it is crucial to reveal the underlying driving forces of geographical variations in animal chemical signals.

Previous studies have demonstrated that habitat-specific ecological selection [3] and genetic drift [4] can lead to rapid changes in chemical profiles between populations, thus facilitating chemical signal divergence. However, unlike acoustic and visual signals, chemical signals are also affected by symbiotic microbiota. According to the fermentation hypothesis for chemical recognition, symbiotic microbes generate metabolites that influence the odour of the host. Population and individual differences in microbe composition direct population and individual differences in odour [5,6]. Although there are numerous examples for geographic variation in chemical signals in insects [7], reptiles [8], birds [9], and mammals [10], the processes by which symbiotic microbes drive the divergence of chemical signal traits, especially in mammals, are still poorly understood.

Bats are the second largest order of mammals and occupy a nocturnal aerial niche. Echolocating bats use acoustic signals, such as social calls and echolocation, for communication, navigation, and prey detection [11]. In addition, these bats require these complicated chemical signals to facilitate social activity [12]. Bats have a wide range in the diversity of their soft-tissue integumentary glands, like many mammals, as well as in the nonglandular odour producing structures that feature both volatile and intensely odorous compounds [13]. These factors make echolocating bats an ideal model system with which to study chemical divergence and its evolutionary driving forces. Nevertheless, few studies have addressed the question of chemical signal divergence of bat odours between populations and their underlying driving forces [14,15,16]. Safi and Kerth (2003) [14] studied female Bechstein’s bats (*Myotis bechsteinii*) in four colonies between 5 and 15 km apart. They found that olfactory signals from the interaural gland secretions were different. Schneeberger et al. (2016) [15] studied male greater sac-winged bats (*Saccopteryx bilineata*) across six colonies (between 100 and 3300 m apart) and reported that the wing-sac chemical “fingerprints” varied between adjacent locations. Exactly how chemical signals vary across a wide range of geographic locations is still not known, however, and even less is known about how symbiotic microbes guide chemical divergence.

**Figure 1:**
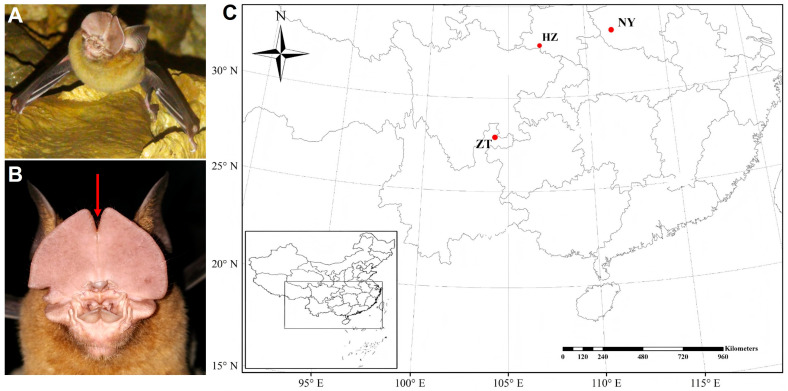
Sampling locations. (**A**,**B**) Adult male Pratt’s leaf-nosed bat, *Hipposideros pratti*. The red arrow indicates the location of the forehead gland. (**C**) Map of sampling localities in China. NY: Nanyang. HZ: Hanzhong. ZT: Zhaotong.

The present study attempted to address these knowledge gaps in Pratt’s leaf-nosed bats (*Hipposideros pratti*) which are found throughout Southeast Asia, including southern China. Male *H. pratti* possess a large sebaceous gland on the forehead that secretes an odorous substance produced by a viscous, tawny-coloured material (Figure 1A,B). Because reproductively mature males secrete these highly odorous substances, it implies that the pheromonal function of these volatile compounds potentially pertains to territorial defence or reproductive behaviour. However, there are few studies on the species’ social and mating systems, and no studies on targeted behavioural or chemical properties of the odorants extruded from the forehead gland.

Here, we examine the fermentation hypothesis in *H. pratti* for the first time. To do so, we first collected forehead gland secretions from three colonies and characterized geographical variation in their chemical profiles. In addition, we investigated the degree to which geographic distribution was responsible for odour variation among the gland’s symbiotic microbiota. We predicted that if odour variation is a consequence of symbiotic microbiota, then the bat gland bacterial community should be significantly correlated with the chemical composition among colonies.

## 2. Materials and Methods

### 2.1. Field Sampling

We visited three colonies in southern China on 10 August 2023, and caught 10 adult male *H. pratti* from each colony (30 bats total; Figure 1C). These colonies include individuals from Nanyang (NY), Hanzhong (HZ) and Zhaotong (ZT) with inter-colony distances as follows: NY-HZ = 460 km, NY-ZT = 938 km and HZ-ZT = 590 km (Figure 1C). Bats were captured after sunset using mist nets positioned at the entrance of their respective roosting caves. The Animal Research Ethics Board of Hebei Normal University China (approval number: HEBTU-2022LLSC044) approved the experiments, which were conducted according to the National Natural Science Foundation of China guidelines for experiments with vertebrate animals. During capture or transport, the bats did not sustain any visible injuries. At the end of the experiments, we released the bats at their original capture sites in good health.

### 2.2. Forehead Gland Secretion Collection and Chemical Compound Analysis

Many males belonging to species of *Hipposideros* in the family Hipposideridae have a more developed forehead gland than do the females. Examples include *H. armiger* [17] and *H. obscurus* [13]. Similarly, the forehead gland of *H. pratti* exhibits sexual dimorphism, being rudimentary and devoid of secretory components in females (CS, pers. observ.). The male forehead gland extrudes a tawny gland substance. We collected this secretion by applying gentle pressure around the gland and transferring the material with presterilized forceps into a 20 mL headspace glass vial (ANPEL, Shanghai, China) fitted with a PTFE-lined septum. Immediately after we captured the bats, we collected the samples. To control for potential environmental or procedural contaminants, one blank sample was collected during each sampling period by exposing the vial to ambient air and waving it three times. Subsequently, we transported the samples to the laboratory where they were stored at −80 °C until needed for analysis.

Before analysis, an internal standard (IS) of 10 µL of 2-octanol (10 mg/L stock in dH_2_O; TCI, Tokyo, Japan) was added to each sample. We heated the mixed samples for 15 min at 60 °C, and then we extracted them using a headspace solid-phase microextraction (SPME) for 30 min. The SPME had a 50/30 µm DVB/CAR/PDMS SPME fibre coating (Agilent, Santa Clara, CA, USA). Following extraction, the volatile compounds were desorbed from the fibre coating. We inserted them directly into the injector port at a temperature of 250 °C.

We used an Agilent 7890 gas chromatograph system with an Agilent 5977 mass spectrometer (Santa Clara, CA, USA) and an EI ion source (70 eV) to conduct the gas chromatography–mass spectrometry (GC-MS) analysis. For the carrier gas, we combined an Agilent DB-Wax capillary column (30 m × 250 µm inner diameter and 0.25 µm thickness) with helium (at 1 mL/min). We injected a 1 µL sample in the 1:1 split mode. We set the purge flow rate to 3 mL/min at the front inlet septum. We maintained a column temperature for 4 min at 40 °C. We increased the temperature at a rate of 5 °C/min until we reached 245 °C, and then we held that temperature for 5 min longer. The temperature of the ion source was 230 °C, front injection temperature was 250 °C, and transfer-line temperature was 260 °C. We performed MS using the full-scan mode in a range from 20 *m*/*z* to 500 *m*/*z*. The solvent delay time was 0 min.

We used Chroma TOF version 4.3X and the National Institute of Standards and Technology (NIST) database to preprocess all chromatographic data [18]. The data included analysis of deconvolution, filtration of the data baseline, measurement of raw peaks, spectrum match, correction for baseline shift, and peak alignment, identification, and integration. We compared volatile compounds with less than 70% similarity with compounds in the NIST database. We excluded relative peak areas less than 0.01% from additional study. We also calculated the relative peak area average for each category of compounds and used the average as the relative peak area for that category. We ran a control blank sample from each colony to ensure that compounds were derived only from the forehead gland secretions and thus could be considered endogenous compounds. We considered compounds to be exogenous compounds or contaminants if the amount of the compounds in the blank sample was higher than or similar to the gland secretion sample.

### 2.3. Bacterial DNA Extraction, Processing and Sequencing

After collecting bat gland secretions, we collected gland microbiota samples from each colony (10 males per colony) by inserting a sterile polyester swab into the forehead gland pocket and rotating the swab five times. We used 1.5 mL sterile tubes with 500 μL of DNA preservation solution (Yuanxin, Shanghai, China) to store the swab tip. We immediately placed all samples on dry ice before we transported to the laboratory. We stored all samples at −80 °C until analysis.

We used the MagPure Soil DNA KF Kit (MD5116, Magen, Guangzhou, China), according to the manufacturer’s guidelines, to extract total genomic DNA from the bacterial swab samples. We employed 1% agarose gels electrophoresis to assess the extracted genomic DNA. We utilized polymerase chain reaction (PCR) for 3 min at 95 °C, followed by 30 cycles for 30 s at 95 °C, for 30 s at 55 °C, and for 45 s at 72 °C (a final extension was 5 min at 72 °C) to amplify the V3-V4 region of the bacteria 16S ribosomal RNA gene. We used primers 338F 5′-ACTCCTACGGGAGGCAGCA-3′ and 806R 5′-GGACTACHVGGGTWTCTAAT-3′. The barcode for each eight-base sequence was unique to each sample. We performed the PCRs three times with a 25 μL mixture with 0.5 μL of FastPfu Polymerase, 1 μL of each primer (5 μM), 1 μL of reverse primer (5 μM), 2.5 μL of 2.5 mM dNTPs, 5 μL of 5 × FastPfu Buffer, and 10 ng of template DNA. We then used the AxyPrep DNA Gel Extraction Kit (Axygen Biosciences, Union City, CA, USA), according to the manufacturer’s instructions, to extract and purify amplicons from 2% agarose gels.

Sequencing libraries were generated using NEB Next^®^Ultra™DNA Library Prep Kit for Illumina (NEB, San Diego, CA, USA) following manufacturer’s recommendations; index codes were added. The library quality (see below) was assessed on the Qubit@ 2.0 Fluorometer (Thermo Scientific, Waltham, MA, USA) and the Agilent Bioanalyzer 2100 system (Nanodrop: 8.06 ± 2.23 ng/μL; range 5.2~12.95 ng/μL). We used an Illumina NovaSeq 6000 PE250 platform (San Diego, CA, USA) to sequence the library and generated 250 bp/300 bp paired-end reads. The Shanghai Origin-gene Biotechnology Co., Ltd. (Shanghai, China) conducted all 16S rRNA sequencing. The NY, HZ, and ZT colonies generated the following: NY generated 1,254,918 raw sequences and the average 125,492 reads per sample ranged from 93,838 to 165,278; HZ generated 1,539,601 raw sequences and the average 153,960 reads per sample ranged from 42,497 to 328,310; and ZT generated 1,811,295 raw sequences and the average 181,130 reads per sample ranged from 139,899 to 251,450.

We used cutadapt v1.2.1 to first remove the adapter sequences that had been added during sequencing [19]. According to a base overlap length of at least 10 bp, we used QIIME v1.9.1 pipeline to form forward and reverse reads into contigs; however, we did not allow any mismatch to occur [20]. We used the following criteria to obtain high-quality reads: no errors in barcode sequence, no presence of ambiguous bases (N), and at least five consecutive high-quality base pairs (Q ≥ 20). We did not allow more than three consecutive low-quality base pairs.

We completed quality processing and obtained 1,107,230 NY samples (average 110,723 reads per sample); 1,326,629 HZ samples (average 132,663 reads per sample); and 1,608,078 ZT samples (average 160,808 reads per sample). According to the UPARSE method, we identified distinct sequences, which we clustered into operational taxonomic units (OTUs). We adhered to a sequence similarity threshold of 97% based and excluded singletons and chimeric sequences [21]. We used the Ribosomal Database Project Classifier to assign taxonomy and used the Greengenes database to obtain taxonomic information for each OTU [22,23]. We used the QIIME pipeline to create phylogenetic tree files. To prevent rare OTUs from interfering and causing errors, we removed OTUs on the basis of <0.001% of the total reads [24]. We also omitted any OTUs classified as chloroplastic or mitochondrial. Finally, we standardized the sampling effort across samples by rarefying the OTU table on the basis of the lowest number of reads (i.e., each sample had 37,599 remaining reads). See Table S1 in the Supplementary Materials for the original OTU data.

### 2.4. Statistical Analyses

We used the peak area of each compound and divided it by the peak area of the internal standard to calculate the relative peak area of the GC-MS peaks. The relative peak area for each compound category was calculated as the average of the relative peak area for each compound category. On the basis of the relative peak area between sample pairs, we calculated the Bray–Curtis similarity index. To perform a nonmetric multidimensional scaling (NMDS) ordination, we used this similarity matrix. To ensure that the relative distance between samples matched the corresponding chemical similarities, we used the NMDS plot to place each sample in a two-dimensional space.

We conducted a univariate one-way analysis of variance (ANOVA) because the number of compounds per individual among the colonies was normally distributed (Shapiro–Wilk tests: *p* > 0.05). We analyzed the difference in the number of compounds in each individual among the three colonies. We conducted a nonparametric analysis of similarity (ANOSIM) to identify if the chemical compounds form the gland secretions varied among the three colonies (see Table S2). With colony as the fixed factor, we used 1000 permutations on the basis of the Bray–Curtis similarity distance matrix. ANOSIM tests feature Mantel-type permutations or employ random programmes that do not make assumptions about date distribution [25]. We defined global *R* as the difference in mean rank within-group dissimilarity relative to group dissimilarity. If global *R* was closer to 1, we determined that the samples from the same colony were more similar to each other than they were to the other colonies. To determine whether categories of compounds of gland secretions (see Table S3) differed between the three colonies, we calculated the Bray–Curtis similarity index based on the mean relative peak area of each category of compounds using 1000 permutations in the ANOSIM test procedure.

To assess whether there were differences in gland bacterial communities between colonies, we first calculated the Shannon diversity, Simpson diversity and Observed richness as measures of alpha diversity using the function “diversity” in the R package (v2.6-4) “vegan” [26]. We performed Wilcoxon signed-rank tests because the Shannon diversity, Simpson diversit and observed richness data were not normally distributed (Shapiro–Wilk tests: *p* < 0.05).

Next, we used Bray–Curtis distance matrices to calculate beta diversity with the function “metaMDS” in the R package “vegan.” We visualized the result with NMDS according to the k = 2 dimensions and completed a permutational multivariate analysis of variance to test the beta diversity between the three colonies. We used the function “adonis” in the R package “vegan” [27]. Because the relative abundance data were not normally distributed (Shapiro–Wilk tests: *p* < 0.05), we compared differences in relative abundances among colonies at the phylum and the genus levels using nonparametric Kruskal–Wallis tests. We used Bonferroni correction to correct *p*-values for multiple comparisons [28].

We selected the Hmisc package based on Spearman correlations to develop cooccurrence networks for bacterial taxa in the three colonies [29]. We selected only genera with a minimum of 30% of the samples for construction of the network to reduce noise and limit false-positive correlations. We retained correlations with *p* < = 0.01 and |*r*| > = 0.6. We used the igraph package to estimate the topological characteristics of the network [30]. If we observed edges only within a subnet, we denoted them as private edges. If we observed edges that were shared among multiple subnets, we defined them as general edges. We determined that keystone taxa (the highly connected taxa) held significance for community function and structure.

We used the “procrustes” function of the R package “vegan” to conduct procrustes analyses. We analyzed the correlation between gland chemical composition and gland microbiota and between chemical category and gland microbiota. We conducted a separate NMDS on the Bray–Curtis dissimilarity matrices for both the gland exudate chemical composition and the gland microbiota, and 9999 permutations were then used to test the significance of the relationship between the two distributions. To assess correlations (Spearman’s *r*) between gland chemical composition distance and gland microbial distance and between chemical category distance and gland microbial distance, we ran two Mantel tests in the “vegan” package [26]. We conducted 10,000 permutations on the basis of Bray–Curtis dissimilarity distance. We used R v4.1.2 for all statistical analyses [31]. We set statistical significance at *p* < 0.05. Because of multiple tests, we employed a Bonferroni correction for *p*-values [32].

## 3. Results

### 3.1. Scent Profiles and Colony-Specific Scent Profile

In total, we detected 579 volatile compounds from 30 individuals across three colonies. After we completed filtering, we selected the 53 volatile compounds still in the dataset for additional analysis (Table S2). We classified the detected compounds into the eight categories: alkene (1.89%), aldehydes (5.66%), alkanes (5.66%), esters (9.43%), ketones (15.10%), aromatic compounds (16.98%), alcohols (22.64%), and carboxylic acids (22.64%).

**Figure 2:**
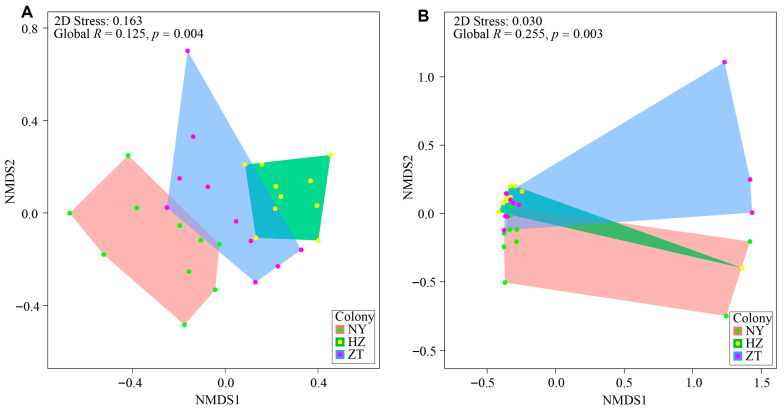
Colony-specific patterns of chemical signals in *Hipposideros pratti* sampled from 10 individuals from each of three colonies. NMDS plots: (**A**) similarity in chemical composition among colonies, and (**B**) similarity of primary categories of chemical compounds among colonies.

There were significant differences in chemical compounds detected in gland secretions among the three colonies based on relative peak area for each compound (Figure 2A). Significant differences were also found in the general categories of compounds between different colonies based on the mean values of the relative peak area of each category of compounds (Figure 2B). This result is retained if we exclude the six outliers in Figure 2B (see Figure S1).

Table 1 and Table S4 provide the total number of volatile compounds according to the profiles of the colonies. We did not observe any significant differences in the number of compounds in each individual among the three colonies (ANOVA: *F*_2,27_ = 0.880, *p* = 0.426). Table 2 provides the coefficient of variation (CV) for each of the eight chemical categories in the frontal glands among and within the three bat colonies. The variation in aldehydes, aromatic compounds and esters among individuals within a colony was less than that among colonies, while the variation in alcohols, alkanes, alkenes and ketones among individuals within a colony was greater than that among colonies. For carboxylic acid, the variation among individuals within a colony was higher than that between colonies only for the SX colony.

### 3.2. Variation in the Gland Microbiota Across Colonies

**Figure 3:**
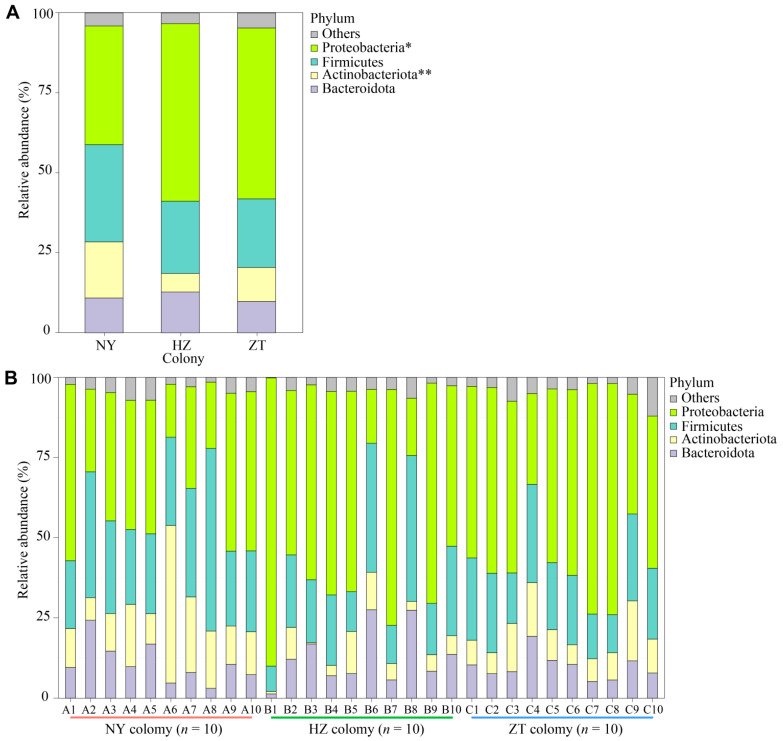
Composition of bacterial communities in the forehead gland microbiota of *Hipposideros pratti* from the Nanyang (NY), Hanzhong (HZ) and Zhaotong (ZT) colonies. (**A**) Relative abundances of primary bacterial phyla (>1% relative abundance) for all three bat colonies. (**B**) Relative abundances of primary bacterial phyla (>1% relative abundance) for all of the samples. Asterisks indicate significant differences in taxa among the three bat colonies. “Others” indicates the sum of the relative abundances of all other phylum or genera levels. * *p* < 0.05, ** *p* < 0.01.

Across all samples from the three colonies, the taxa at the phylum level were dominated by Proteobacteria (PR; ~48.69%), Firmicutes (FI; ~24.81%), Actinobacteriota (AC; ~11.32%) and Bacteroidota (BA; ~11.13%) (Figure 3A). We observed significant variation in the relative abundance of AC and PR among the three bat colonies (Kruskal–Wallis; PR: χ^2^ = 7.347, *p*  =  0.025; AC: χ^2^ = 12.003, *p* = 0.002; Figure 3A,B), but no differences in the relative abundance of FI and BA between colonies (Kruskal–Wallis; FI: χ^2^ = 5.234, *p*  =  0.073; BA: χ^2^ = 3.343, *p*  =  0.842).

**Figure 4:**
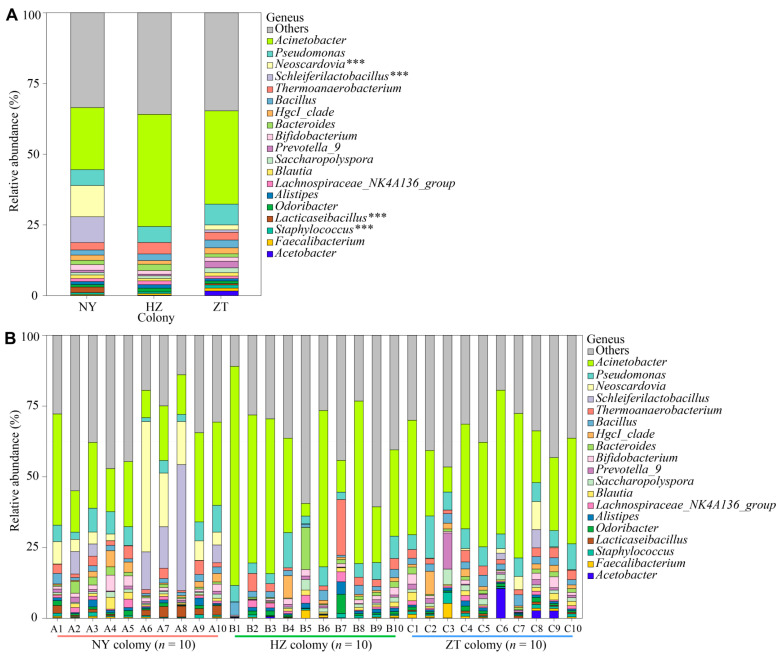
Composition of bacterial communities from the Nanyang (NY), Hanzhong (HZ) and Zhaotong (ZT) colonies in the forehead gland microbiota of *Hipposideros pratti*. (**A**) Relative abundances of primary bacterial genera (>1% relative abundance) for all three bat colonies. (**B**) Relative abundances of primary bacterial genera (>1% relative abundance) for all of the samples. The asterisks represent taxa with significant differences between colonies. “Others” indicates the sum of the relative abundances of all other phylum or genera levels. *** *p* < 0.001.

The taxa at the genus level were dominated by *Acinetobacter* (~31.53%), *Pseudomonas* (~6.20%), *Neoscardovia* (~4.27%), *Schleiferilactobacillus* (~3.34%), *Thermoanaerobacterium* (~3.11%), *Bacillus* (~2.32%), *HgcI_clade* (~1.74%), *Bacteroides* (~1.67%), *Bifidobacterium* (~1.56%), *Prevotella_9* (~1.23%), *Saccharoolyspora* (~1.20%), *Blautia* (~1.10%), *Lachnospiraceae_NK4A136_group* (~1.09%), *Alisipes* (~1.07%) and *Odoribacter* (~1.04%) (Figure 4A).

As shown in Figure 4A,B, we also observed significant differences in the relative abundance of *Neoscardovia*, *Schleiferilactobacillus*, *Lacticaseibacillus* and *Staphylococcus* between colonies (Kruskal–Wallis; all *p* < 0.001; χ^2^ = 17.686–21.786). Also shown in Figure 4A,B, we did not identify any significant differences in the relative abundances of *Acinetobacter*, *Pseudomonas*, *Thermoanaerobacterium*, *Bacillus*, *HgcI_clade*, *Bacteroides*, *Bifidobacterium*, *Prevotella_9*, *Saccharopolyspora*, *Blautia*, *Lachnospiraceae_NK4A136_group*, *Alistipes*, *Odoribacter*, *Faecalibacterium* and *Acetobacter* (Kruskal–Wallis; All *p* > 0.05; χ^2^ = 0.258–5.582).

**Figure 5:**
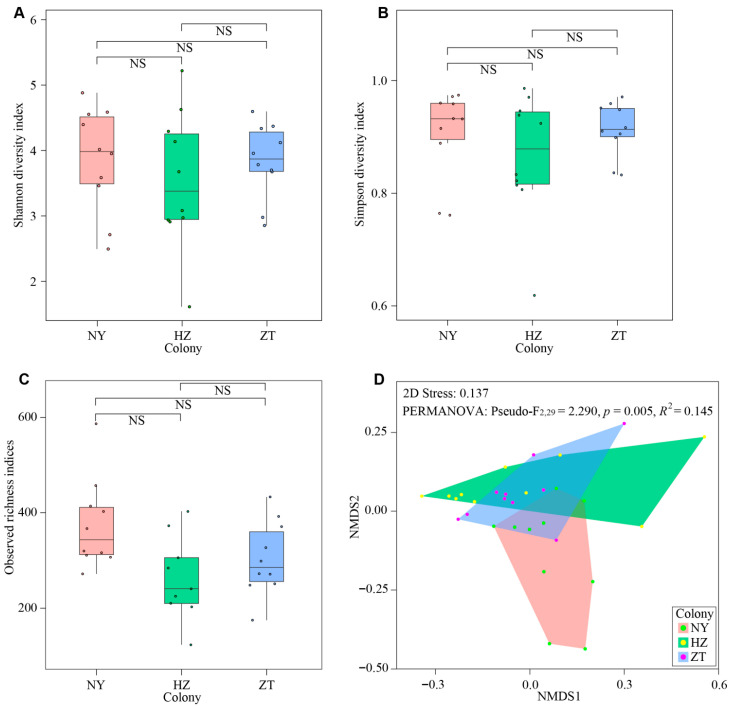
Diversity of bacterial communities in the forehead gland microbiota of *Hipposideros pratti* from the Nanyang (NY), Hanzhong (HZ) and Zhaotong (ZT) colonies. Comparison of Shannon diversity (**A**), Simpson diversity (**B**) and Observed richness (**C**) across colonies. (**D**) NMDS plots according to Bray–Curtis dissimilarity matrices reveal bacterial community structure among the three bat colonies. NS, not significant.

Wilcoxon signed-rank tests indicated that no significant differences could be found in Shannon diversity, Simpson diversity and Observed richness between NY and HZ colonies, NY and ZT colonies, or HZ and ZT colonies (All *p* > 0.05; Figure 5A–C). We conducted NMDS analysis according to the Bray–Curtis dissimilarity matrices and, in contrast, we observed a significant difference in the community structure of the gland bacterial of the bats in the three colonies (NMDS with stress = 0.137, RMANOVA, Pseudo-F_2,29_ = 2.290, *p* = 0.005, *R*^2^ = 0.145; Figure 5D). Thus, alpha diversity was similar between colonies, but the species composition differed significantly between colonies.

**Figure 6:**
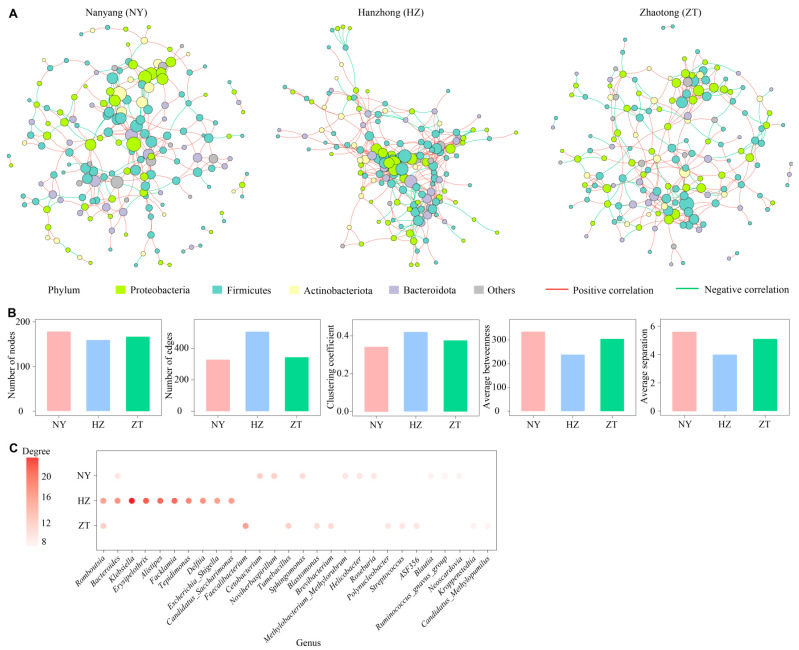
Cooccurrence networks of bacterial communities from the Nanyang (NY), Hanzhong (HZ) and Zhaotong (ZT) colonies in the forehead gland microbiota of *Hipposideros pratti*. (**A**) Phylum-level cooccurrence networks for bat gland bacteria. Red edges are positively correlated and green edges are negatively correlated. Nodes that belong to the same phylum are the same colour. (**B**) Topological characteristics for the bacterial cooccurrence networks. (**C**) The top 10 key taxa in each colony are indicated by colour, and each point represents the average degree of taxon. Taxa that are not key taxon are indicated in the blank positions.

To construct microbial cooccurrence networks, we integrated genus-level interactions within the bat gland bacterial communities across the three colonies. We observed that topological characteristics of these networks were variable among the colonies (Figure 6A). The NY colony network indicated a higher number of nodes (*n* = 178) than the HZ and ZT colony networks. In contrast, the HZ network demonstrated a greater number of edges (*n* = 505) than the NY and ZT colonies. The HZ network had a higher clustering coefficient value (0.42) than the ZT network (0.38) or the NY network (0.34). Moreover, the HZ network exhibited a lower value of average betweenness (238.9), while the NY (334.8) and ZT (304.7) networks had higher values of average separation (Figure 6B). We analyzed the keystone taxa among the three cooccurrence networks. Figure 6C shows the top 10 nodes that demonstrated the highest degree values for all of the networks (28 genera). We identified *Faecalibacterium* in the ZT network, *Klebsiella* in the HZ network, and *Noviherbaspirillum* in the NY network as the keystone taxa. *Bacteroides* and *Romboutsia* were the keystone genera for more than one network.

### 3.3. Relationships Between the Gland Microbiota and Chemical Composition

**Figure 7:**
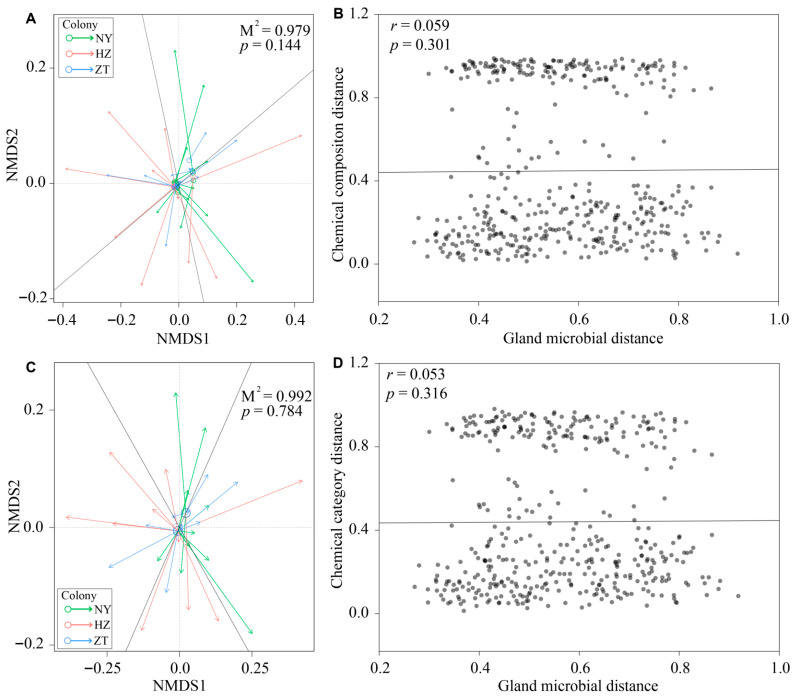
Relationship between chemical signals and gland microbiota of *Hipposideros pratti* from the Nanyang (NY), Hanzhong (HZ) and Zhaotong (ZT) colonies. Procrustes analysis of the correlation between gland bacterial communities and the chemical compositions (**A**) and chemical categories (**C**) based on Bray–Curtis dissimilarity matrices. The base of the arrows represents NMDS coordinates for the gland bacterial communities and the arrow heads point to the NMDS coordinates for the gland chemical composition and chemical categories. Mantel tests of the correlation between gland bacterial communities and the chemical compositions (**B**) and chemical categories (**D**) based on Bray–Curtis dissimilarity matrices.

As shown in Figure 7A,C, according to the procrustes analysis, the bat gland bacterial community’s structure did not have a significant correlation with chemical composition (PROTEST: *r* = 0.143, *p* = 0.144) or with chemical categories (PROTEST: *r* = 0.090, *p* = 0.784) among the three colonies. A Mantel test showed that the pairwise gland microbial distance was not significantly correlated with pairwise gland chemical composition distance (Spearman’s *r* = 0.059, *p* = 0.301; Figure 7B) and with pairwise gland chemical category distance (Spearman’s *r* = 0.053, *p* = 0.316; Figure 7D).

## 4. Discussion

In this study, chemical signals from the forehead gland secretions of male *H. pratti* showed significant geographical variation across three colonies in southern China. However, chemical divergence was not significantly correlated with the forehead gland microbiota, suggesting that variation in symbiotic microbe communities may not result in divergence of chemical signals across colonies.

### 4.1. Variation in Chemical Signals

The categories and concentrations of chemical compounds among the three bat colonies has observed differences. Our findings indicated that the forehead gland secretions of *H. pratti* had odours that conveyed identity information at the colony level. The variations in the chemical signals among the three colonies within a region were significant, perhaps demonstrating the presence of “odour dialects,” which are analogous to vocal dialects [17,33]. These odour dialects could be used to identify colonies and may make it easier to recognize intruders from colonies that are not local. This may be important in our system because a recent study based on mitochondrial DNA demonstrated that the Nanyang (NY), Zhaotong (ZT) and Hanzhong (HZ) colonies of *Hipposideros pratti* may belong to a single genetic lineage found in central western China [34]. As such, there may be no genetic colony-level markers that could indicate a colony-specific identity. Liu et al. (2024) described the high-rate gene flow among regional colonies [34], which indicated that interactions between conspecifics from different colonies were frequent. Therefore, the ability to precisely recognize intruders from foreign colonies could enable divergence in these chemical signals among regional colonies. Other studies have verified that bats can recognize individuals on the basis of their odours [16,35]. However, future behavioural experiments are needed to determine whether male *H. pratti* have the ability to discriminate colony members from non-members based on odour cues, and whether female bats preferentially respond to odours of local versus non-local males.

As shown in Table 2, we determined variation in geography in the chemical signals of *H. pratti* across a wide geographic range, but most of this variation was attributed to individual differences within the colonies. This high variation among individuals in categories of chemical compounds revealed that the scent of the secretions from the forehead gland of *H. pratti* likely contributes to individual recognition. Individual recognition or discrimination on the basis of forehead gland odours could be significant for male *H. pratti*, most notably in dark caves. For example, conflicts between strangers may be more intense than those between nearby neighbours, a phenomenon termed the ‘dear enemy effect’ [36,37]. Physical fighting (i.e., boxing and wrestling) is costly to bats. Therefore, being able to distinguish between neighbours and strangers using forehead gland odours may make it easier for males to assess threat levels and their opponents’ fighting ability. As a result, males can better determine whether or not to continue competing. Additional testing is needed to determine whether or not male *H. pratti* can discriminate between individuals on the basis of forehead gland odours.

### 4.2. Variation in Gland Microbiota

We found no significant gland microbial difference in alpha diversity across colonies (Figure 5A–C). The Shannon diversity index integrates species richness (the number of bacterial taxa) and evenness (the proportional abundance balance of each taxon), while the Simpson diversity index emphasizes the dominance of abundant taxa, and Observed richness directly reflects the total number of bacterial taxa detected. The consistent absence of inter-colony differences across these metrics collectively implies that the “number of bacterial species”, the “equitability of their relative abundances”, and the “dominance pattern of dominant taxa” in the forehead glands are highly similar across the three geographic colonies of *Hipposideros pratti*. In contrast, we found a significant gland microbial difference in beta diversities across colonies (Figure 5D). Beta diversity measures the dissimilarity of community composition between colonies. The significant differences in beta diversity suggest that the species composition and/or their relative abundances vary notably across the three colonies. Those differences could be related to the geomorphology of the caves, reflecting differences in cave volume, colony size, temperature or humidity. According to studies on *Myotis lucifugus*, a significant reason for patterns found in skin microbial communities is the local environment [38]. The forehead gland of male *H. pratti* is not completely closed and some glandular hairs and forehead secretions are often exposed to the air. As a result, gland bacterial community structure of *H. pratti* may be influenced by the local cave environment.

We found that the co-occurrence networks of the HZ colony had more nodes and edges (Figure 6A,B), indicating the bacterial network of the HZ colony is more complex and tightly connected than the other colonies. In these networks, other microbial taxa are not as important as keystone taxa, and the collapse of the network or modules may be triggered by their loss [39]. There was minimal overlap in keystone taxa across the three colonies (Figure 6C). This result indicated that the keystone taxa and interaction patterns of the three colonies are colony-exclusive, reflecting functional differentiation of microbiota driven by geographic isolation.

### 4.3. What Drives Variation in Chemical Signals?

According to the fermentation hypothesis, symbiotic microbes inside odour glands create metabolites that have an impact on the scent profile of the host. Colony and group differences in the microbe composition, in turn, drive colony and group differences in odour [5]. For instance, in hyenas, bacterial taxa covary with population-specific odour profiles, which lends support to the fermentation hypothesis of bacterially mediated chemical communication [40,41]. The male forehead gland of *H. pratti* exhibits a saccular structure penetrated by a pen-like long hair, with fleshy lobular protrusions on both sides. The glandular tissue of *H. pratti* is closely connected to the skin surface, forming specialized channels for storing and releasing secretions. This specialized channel would appear to offer a warm, moist and semi-anaerobic microenvironment that could support a thriving community of symbiotic microbes. As a result, we predicted that symbiotic microbes inhabiting odour glands should influence the chemical profiles in *H. pratti*. However, although we found significant differences in both chemical profiles and the gland bacterial community structure among the NY, HZ and ZT colonies, Procrustes analysis and Mantel tests revealed no significant correlation between bat gland microbiota and gland chemical composition. These results suggest that bat gland microbiota may not contribute to gland chemical cues as predicted by the fermentation hypothesis, at least not for *H. pratti*. One possible explanation is that microbial biomass within the gland may be too low, or that the microbes may exist in a metabolically inactive state, limiting their ability to influence the chemical composition of the gland exudate. Their metabolic byproducts would thus be present at concentrations too low to override or modify the host’s intrinsic chemical signature. The function of gland microbiota in *H. pratti* should be explored in future studies. However, we should be cautious of assuming that bat gland microbiota may not contribute to gland chemical cues. This is because although the microbial community compositions of the three colonies exhibit significant differences, functional redundancy may exist (e.g., producing identical or similar volatile organic compounds). Consequently, the observed “absence of a significant correlation between variations in microbial taxonomic composition and differences in chemical signals” is reasonable.

## 5. Conclusions

This study demonstrated that the forehead gland scents of male *H. pratti* exhibit significant geographical variation across three colonies. However, the symbiotic microbe community inhabiting the forehead gland may not be a potential force driving chemical variation between colonies. We identified two limitations in this study. First, the geographical variation in chemical signals is that the relationship between stochastic processes and chemical divergence [4], and the relationship between ecological selection and chemical divergence [3] were not addressed. Future studies should also consider the relative importance of ecological and genetic sources for chemical divergence within and between species from a diversity of taxa. Second, the current study lacks direct evidence that connects specific microbial metabolic functions to particular odour compounds. Future research should integrate metagenomics and metabolomics to more directly test whether microbial functions covary with the geographical variation in chemical signals. Additional testing to examine how broad-spectrum antibiotics may be applied to the forehead gland should verify the causal relationship between the host’s odour profiles and the odour gland’s bacterial community structure.

## Figures and Tables

**Table 1:** Description of the total number of gland secretion compounds in each colony of *Hipposideros pratti.*

Colony	Mean Number of Compounds per Individual ± SE *	Total Number of Compounds Occurring in All Samples from the Colony	Number of Samples
Nanyang (NY)	41.70 ± 2.69	9	10
Hanzhong (HZ)	37.50 ± 1.45	17	10
Zhaotong (ZT)	39.00 ± 1.45	16	10

* SE: standard error.

**Table 2:** The coefficient of variation (CV) of eight chemical categories of compounds in *Hipposideros pratti* within and between colonies. NY: Nanyang. HZ: Hanzhong. ZT: Zhaotong.

	Alcohol	Aldehyde	Alkane	Alkene	Aromatic Compound	Carboxylic Acid	Ester	Ketone
Inter-individual CV (%; NY)	66.3	51.7	102.4	202.7	49.3	79.0	83.6	56.4
Inter-individual CV (%; SX)	38.0	92.5	74.7	68.2	31.0	94.7	96.7	88.0
Inter-individual CV (%; ZT)	72.8	60.2	64.4	215.8	37.0	70.1	55.5	68.6
Inter-colonial CV (%)	25.4	110.2	48.6	66.0	51.3	79.7	98.9	31.7

## Data Availability

The data on which this study is based are available in the Supplementary Material.

## Supplementary Materials

The following supporting information can be downloaded at: https://www.mdpi.com/article/10.3390/biology15020114/s1. The four supplementary tables and one supplementary figure are as follows: Table S1. Taxonomy and OTU information for the samples. Table S2. Peak area ratios for the compound compositions in each sample. We divided the peak area of each compound by the area of the internal standard. Table S3. Peak area ratios for the compound categories in each sample. We divided the peak area of each compound by the area of the internal standard. Table S4. Sample compounds found in each colony. Figure S1. Chemical signals for colony-specific patters in *Hipposideros pratti*. We sampled 10 individuals from all three colonies. According to the NMDS plots, we observed similarities among colonies in the primary categories of the chemical compounds.

## Abbreviations

The following abbreviations are used in this manuscript:

NY	Nanyang
HZ	Hanzhong
ZT	Zhaotong
GC-MS	gas chromatography–mass spectrometry
NMDS	nonmetric multidimensional scaling
ANOSIM	nonparametric analysis of similarity
