# Supplementary figures and images for: Do Symbiotic Microbes Drive Chemical Divergence Between Colonies in the Pratt’s Leaf-Nosed Bat, Hipposideros pratti?
Source: Biology (Basel). 2026 Jan 6;15(2):114. doi: 10.3390/biology15020114 (PMC12837207; doi:10.3390/biology15020114)

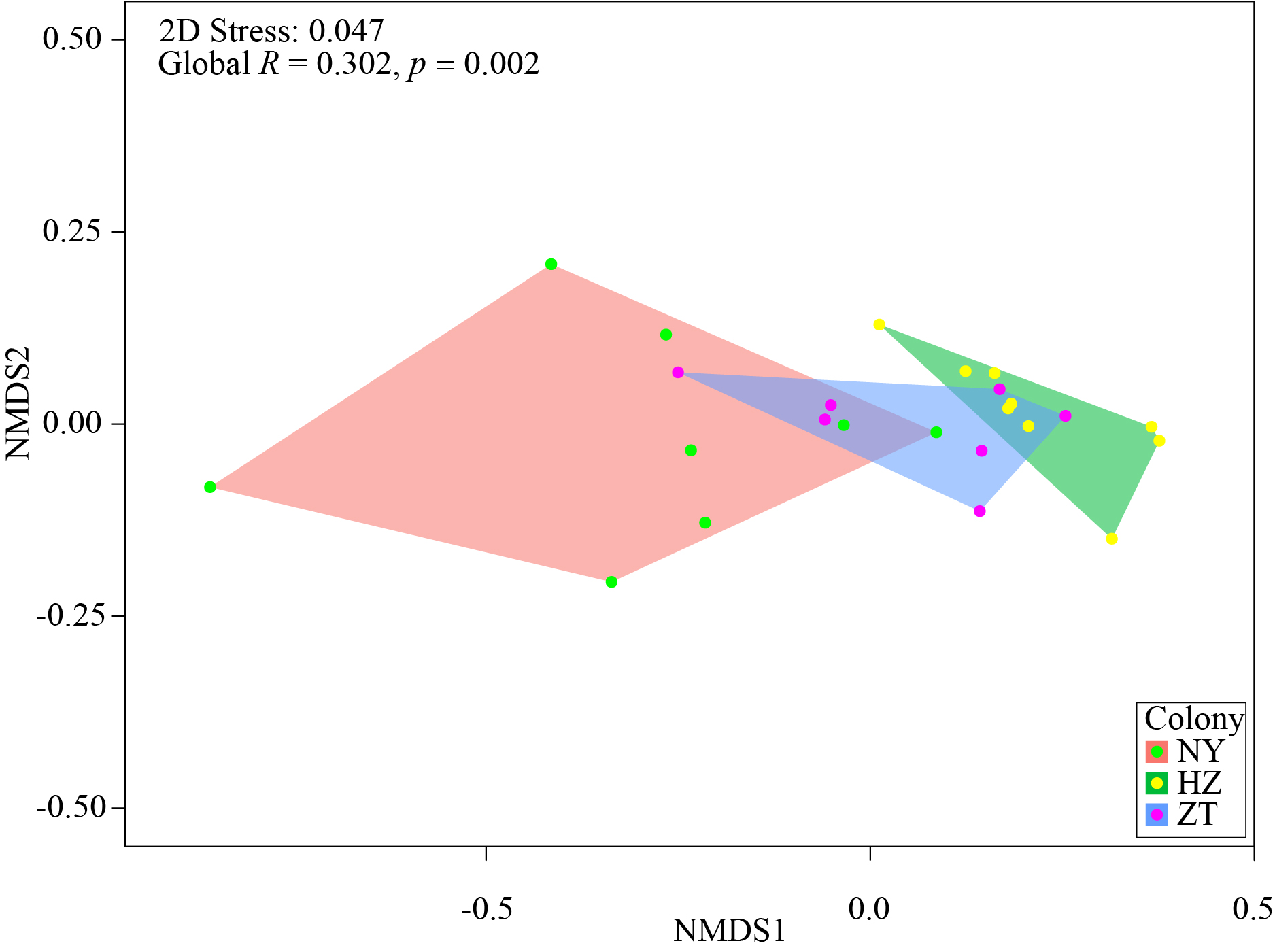

Supplement: Supplementary file 1 [file biology-15-00114-s001.zip › Supplementary Files/Figure S1.jpg]
